# Design and Fabrication of a Tunable Optofluidic Microlens Driven by an Encircled Thermo-Pneumatic Actuator

**DOI:** 10.3390/mi13081189

**Published:** 2022-07-28

**Authors:** Wei Zhang, Heng Li, Yongchao Zou, Pengpeng Zhao, Zeren Li

**Affiliations:** 1 Shenzhen Key Laboratory of Ultraintense Laser and Advanced Material Technology, Center for Advanced Material Diagnostic Technology, College of Engineering Physics, Shenzhen Technology University, Shenzhen 518118, China; lizeren@sztu.edu.cn; 2 MOE Key Laboratory of Material Physics and Chemistry under Extraordinary Conditions, and Shaanxi Key Laboratory of Optical Information Technology, School of Physical Science and Technology, Northwestern Polytechnical University, Xi’an 710129, China; 3 Nanophotonics Research Center, Shenzhen Key Laboratory of Micro-Scale Optical Information Technology & Institute of Microscale Optoelectronics, Shenzhen University, Shenzhen 518060, China; 2060493021@email.szu.edu.cn; 4 College of Meteorology and Oceanography, National University of Defense Technology, Changsha 410073, China; yongchao.zou@foxmail.com; 5 Innovation Center for Smart Medical Technologies and Devices, Binjiang Institute of Zhejiang University, Hangzhou 310053, China; zhaopengpeng@zju-bj.com

**Keywords:** optofluidic lens, thermo-pneumatic, 3D printing

## Abstract

This paper presents the design, simulation, fabrication, assembly, and testing of a miniature thermo-pneumatic optofluidic lens. The device comprises two separate zones for air heating and fluid pressing on a flexible membrane. A buried three-dimensional spiral microchannel connects the two zones without pumps or valves. The three-dimensional microfluidic structure is realized using a high-resolution three-dimensional printing technique. Multi-physics finite element simulations are introduced to assess the optimized air chamber design and the low-temperature gradient of the optical liquid. The tunable lens can be operated using a direct-current power supply. The temperature change with time is measured using an infrared thermal imager. The focal length ranges from 5 to 23 mm under a maximum voltage of 6 V. Because of the small size and robust actuation scheme, the device can potentially be integrated into miniature micro-optics devices for the fine-tuning of focal lengths.

## 1. Introduction

The optofluidic lens has attracted considerable attention in the fields of miniature imaging systems [1], biomedical instruments [2], and three-dimensional (3D) vision systems [3]. Unlike conventional optical systems with glass materials, the optofluidic lens is adjusted by tuning the liquid lens shape [4,5] or changing the liquid-crystal refractive index [6] without sophisticated mechanical transmission structures. The use of the optofluidic lens is thus helpful in simplifying optical systems [7] and controlling optical power precisely [8]. Compared to the solid microlens, the optofluidic lens provides the flexibility of controllable focal length, highly smooth optical surfaces, and miniaturized and integrated packaging.

The variable-shape optofluidic lenses can be classified into two categories, namely liquid–membrane lenses and liquid–liquid lenses. In the case of a membrane lens, the lens comprises a cylindrical chamber sandwiched between an elastic membrane and a transparent substrate. The membrane is deformed when pressure is applied to the liquid, thus shaping the lens profile. In contrast, liquid–liquid lenses generally rely upon the dielectric or electro-wetting properties of the liquid. Both electro-wetting [9] and liquid dielectric lenses [10] use external voltage to control the shape of liquid and are available commercially; however, high voltages are typically required in liquid lenses. Furthermore, fast focusing using a pinned-contact-line liquid lens has been reported [11], but it creates more challenges for an integrated packaging.

According to the relative orientation of the light path and the lens substrate, optofluidic lenses can be categorized as in-plane or out-of-plane liquid lenses [12]. In-plane liquid lens is the light propagating in the plane of the device substrate. Compared with out-of-plane lenses, in-plane lenses present higher integration capability with the microfluidic chip for lab-on-a-chip applications [13,14,15,16]. The optical interface is formed between a liquid core and liquid cladding with different refractive indices and laminar flow speeds. These lenses show the advantages of a high degree of complexity, such as changing the lens shape from convex to concave or from planar convex to the biconvex lens. For out-of-plane liquid lenses, such as liquid–membrane lenses, the light path is perpendicular to the plane or lens surfaces. Liquid lenses are compatible with conventional optical systems and could replace fixed-glass lenses. Through the deformation of the liquid lens surface, a parallel light beam can be focused at a spot on the optic axis of the optical system, so out-of-plane liquid lenses are widely used for the imaging system. The architecture has been found in liquid lens systems for endoscopes [17], digital camera lenses, microscopes, and mobile phone lenses [18].

We propose the design of a miniature optofluidic lens with a soft membrane, because membrane lenses have the advantages of a faster execution time, greater design freedom [19], and easy implementation of actuation mechanisms. The membrane curvature is varied by manipulating a small volume of liquid into the liquid chamber, and an efficient actuation mechanism is thus urgently required in practice [20]. Various actuation mechanisms have been used for tunable membrane lenses. Pneumatic actuation using compressed air is a simplified approach used to control the liquid volume. This method is easily implemented and can quickly demonstrate the lens’s functionalities. However, an external air pumping system and extra tubing, which might be bulky and impracticable for the integration system, are essential for liquid lens operation [21]. A piezoelectric actuation scheme is adopted for actuating the displacement of membranes but requires relatively complex driving circuits [22]. Additionally, it is difficult to obtain high-quality thick films of lead zirconate titanate for large deformation, which can make the lens system bulky [23]. Magnetic actuation has the characteristics of precise positioning and a short response time but requires a complex external structure for handling rotary motions [24]. Electrostatic actuation ensures a compact package but requires relatively high driving voltages [25].

Thermo-pneumatical liquid lenses have been studied in recent years. Among other actuation mechanisms, thermally driven devices have outstanding advantages of great force, simple actuation scheme, and low driving voltage [26,27,28]. On-chip thermal actuation is easily obtained by structuring metal layers and miniaturizing the structure of the lens [29]. The adoption of a microchannel chip relies on Micro-Electro-Mechanical-System (MEMS) fabrication technologies that mold microchannels on a photolithographic resist and then seal the open channels with a flat substrate and finally create channels with rectangular cross-sections. The fabrication requires a cleanroom environment and is time-consuming. Meanwhile, the lens design is limited to the fabrication of a 3D structure, as the channels will interlock with each other for a two-dimensional structure [30,31,32]. The process of 3D printing can fabricate truly 3D microchannel networks in a single step from a computer model without requiring a cleanroom environment. The process of 3D printing fabricates structures with variable heights to achieve the device package in one step without involving any alignment or sacrificial parts. It successfully solves the issue of liquid leakage, because the microchannels are embedded in the structure. The combination of the thermal-pneumatic actuation and the 3D design means that the optofluidic lens has great potential in numerous applications, enabling focusing on objects located at different distances from the photographing device, such as a smartphone camera.

In this paper, we present a self-contained, integrated tunable optofluidic lens with an encircled thermal-pneumatic actuator. Tunability is achieved by heating the air encapsulated in the thermal chamber. As the air expands, the optical liquid flows through a 3D spiral microchannel to the liquid chamber, and the membrane deforms. A high-resolution 3D-printing technology is adopted for the optofluidic chip. A thermal-pneumatic-mechanical analysis is conducted to estimate the membrane deformation for the allowable temperature change, and multi-physics simulations are conducted to assess the device’s temperature homogeneity, especially for the low-temperature gradient in the optical liquid. The combination of the thermal-pneumatic actuation and the 3D architecture design means that the optofluidic lens has great potential in numerous applications requiring the fine-tuning of focal lengths.

## 2. Design and Analysis

### 2.1. Lens Concept

**Figure 1:**
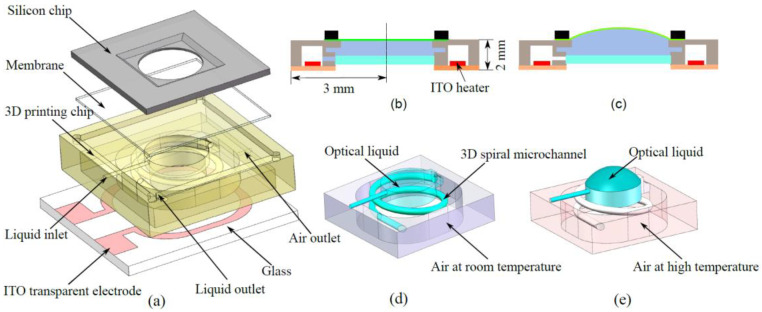
(**a**) The 3D design of the membrane–liquid lens. (**b**) Cross-section of the lens. The thin film is sandwiched between the silicon lens chip and the 3D-printed microfluidic chip. The dimensions are 6 × 6 × 2 mm^3^. (**c**) Pressure actuation generated on-chip using the Joule effect. (**d**) Detailed view of the liquid fields. At room temperature, the liquid chamber and spiral microchannel are filled with silicone oil, and sealed air surrounds the optical liquid. (**e**) At high temperature, the air volume expands, and the liquid in the spiral channel moves into the liquid chamber, finally deforming the membrane.

Figure 1a is a schematic of the optofluidic lens with a closed 3D fluidic system. The liquid lens comprises four elements: a flexible polyacrylate membrane, a silicon chip (∅=2 mm), a 3D-printing microfluidic device, and transparent indium tin oxide (ITO) glass. The liquid lens has a full size of $6\times 6\times 2{\;\mathrm{mm}}^{3}$ and a circular opening (∅=2.1 mm) at its center. There are two straight microchannels in the microfluidic chip, one on the top surface for filling the lens cavity with the optical liquid and the other on the sidewall as a vent for removing air. Silicone oil (having viscosity of 10 cSt and a refractive index of 1.40) was used as the optical liquid. During the observation at room temperature for 240 h, no change in the weight of the lens device was observed, owing to the low vapor pressure of the silicone oil. The oil was inserted with a syringe (having a diameter of 0.15 mm) into the liquid inlet (diameter: 0.2 mm). Air bubbles were removed from the air outlet.

As shown in Figure 1b,c, the position of the liquid/air interface meniscus is stable when there are equal forces in the two cavities, such that the membrane is flat and without optical power. When a voltage is applied to the heater structure, the air temperature increases through Joule heating, and the air volume expands. The optical liquid can be assumed to be incompressible, and the volume change of the air thus deforms the membrane. This produces an upward actuation to form a convex shape. Figure 1d,e shows the air and optical liquid. The liquid chamber is directly connected to the air cavity by the 3D microchannel network without a pump membrane. The overall microchannel structure is designed to be a spiral bridge, so that the circular sections vertically overlap without horizontal intersections.

### 2.2. Lens Design

In the paraxial approximation for a thin plano-convex lens, the focal length is f=R/(n−1), where R is the radius of the membrane curvature and n is the refractive index of the liquid. For a given liquid, the focal length is defined by the radius of curvature. A spherical surface having a radius of curvature R is desired to form a liquid lens. One can apply standard spherical lens equations to deal with the liquid lens if the altitude of the lens surface satisfies the relation h≪r [24], where r is the radius of the liquid lens aperture.

**Figure 2:**
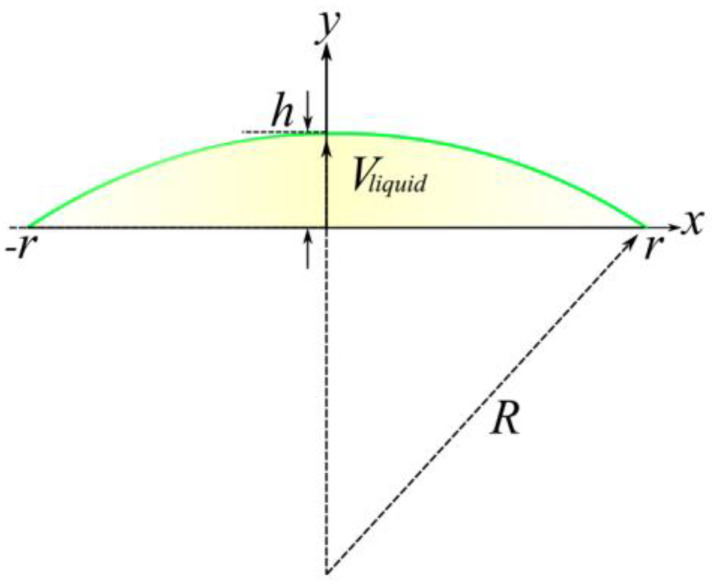
Approximation of the membrane lens profile by a spherical cap.

It is evident from Figure 2 that $R^{2}={\left(R-h\right)}^{2}+r^{2}$, for the typical lens with h≪r, and hence, $R\approx r^{2}/2h$. The lens focal length is

(1) $$f=\frac{r^{2}}{2h\left(n-1\right)},$$

The membrane displacement has the shape of a spherical cap, and the volume of the liquid is thus

(2) $$\Delta V_{liquid}=\frac{1}{3}\pi h^{2}\left(3R-h\right),$$

By eliminating *R*,

(3) $$\Delta V_{liquid}=\frac{1}{2}\pi hr^{2}-\frac{1}{3}\pi h^{3}\approx \frac{1}{2}\pi hr^{2},$$

Finally, the focal length is determined from the variable liquid volume as

(4) $$f=\frac{\pi r^{4}}{4\Delta V_{liquid}\left(n-1\right)},$$

The critical limitation of the above analytical formulas is that the real shape of the membrane is not precisely spherical but aspherical with vertex curvature. However, the formulas are useful for the first estimation of the focal length and the injected fluidic volume under hydrostatic pressure.

One must decide the correct volume of the air chamber when designing the thermo-pneumatic actuator for the lens. The volume of the air-trapping chamber $V_{0}$ should be small enough for the device to be compact but large enough to expand by $\Delta V_{air}$ without heating to an extreme temperature. Here, we have combined the ideal gas relationship $\left(PV/T=NK_{B}\right)$ and a multi-physics simulation to estimate the air chamber volume.

### 2.3. Thermo-Pneumatic Actuator Design

#### 2.3.1. Thermo-Mechanical Model

**Figure 3:**
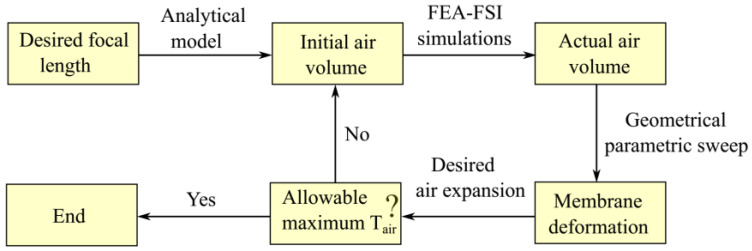
Flow chart of the air volume optimization.

A strategy combining the analytical model and FEA simulation was developed to find the optimized air volume according to the allowable temperature. Figure 3 presents a detailed design flow chart. First, the initial air volume was calculated using the isobaric heating pressure equation. Second, the dynamic fluid–structure interaction (FSI), heat transfer in solids and fluids, and mechanics of solids were combined to analyze the model in a multi-physics simulation in COMSOL 5.4. Third, a geometrical parametric sweep of the air volume was performed through a specified range from the initial air volume to trade off the air volume and maximum allowable temperature.

**Figure 4:**
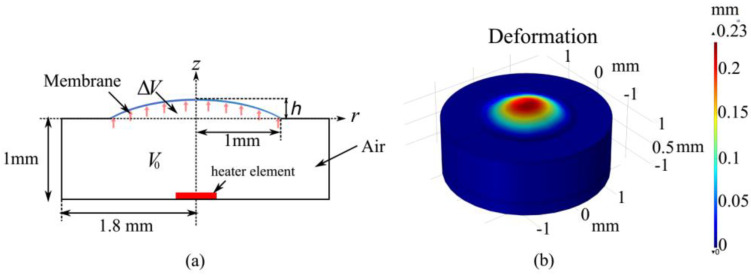
(**a**) Cross-section of the simulation model. A uniformly loaded membrane (2 mm in diameter) is clamped on the top. (**b**) Simulation results of membrane deformation when a temperature of 40 °C is used as a boundary condition.

**Figure 5:**
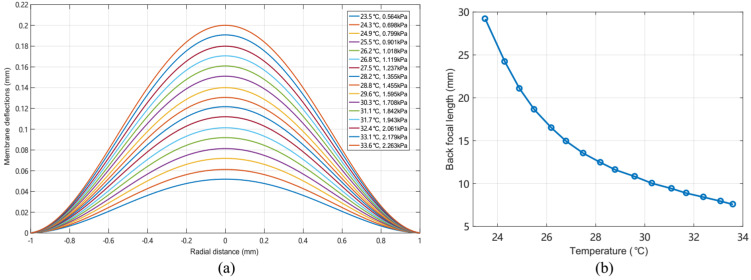
(**a**) Simulated membrane profiles of the membrane for an applied temperature ranging from 23.5 to 33.6 °C. (**b**) Focal lengths calculated according to the membrane profiles.

As depicted in Figure 4a,b, the simplified 3D cylindrical model accounted for the membrane deformation according to the temperature variation. The flexible membrane (having thickness of 0.1 mm, density of $960\;\mathrm{kg}/m^{3}$, a Young’s modulus of 1.5 MPa, and Poisson’s ratio of 4.9) was adopted in the model. The air inside the cavity expands when it is heated, and the membrane is displaced by the increasing pressure. The pressure drop in the liquid flow in the microchannel is negligible, because the use of a low-velocity optical liquid results in a low flow resistance and small pressure drop according to Hagen–Poiseuille’s law [33]. The initial air volume is finally obtained as 10.18 μL. The maximum allowable temperature is limited to 40 °C for the sealed air (whereas the room temperature is 20 °C). Figure 5 shows the simulated membrane profiles for the different temperatures and corresponding pressures. The focal lengths are calculated for different altitudes of the membrane using Equation (1).

#### 2.3.2. Electro-Thermal Model

**Figure 6:**
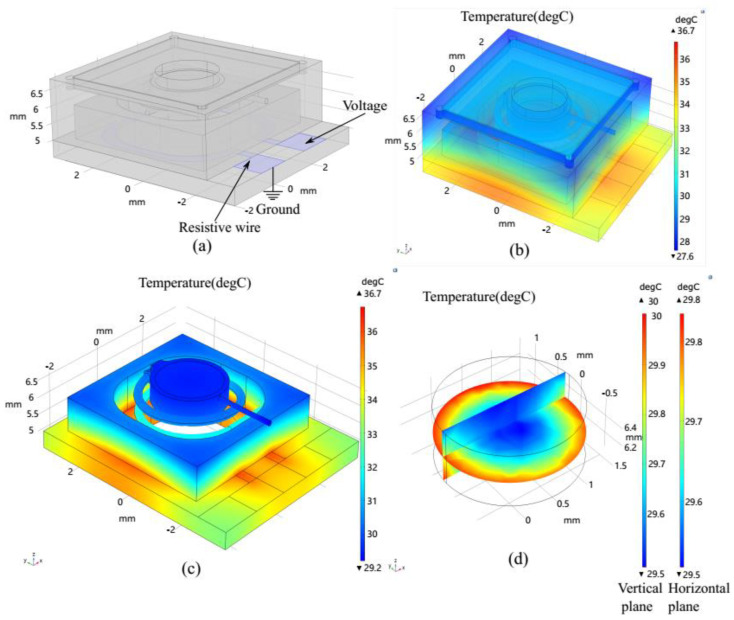
Simulation results of the temperature distribution of the proposed device. (**a**) FEM model of the optofluidic lens. For the resistive wire, a 150-nm-thick ITO layer was considered as a two-dimensional structure on the glass substrate. (**b**) Temperature distribution of the complete device for a bias of 5 V applied to the wire. (**c**) The temperature distribution of the fluidic fields. (**d**) Temperature gradient of the optical liquid in the horizontal and vertical directions.

A low temperature gradient in an optical system is essential, because the properties of optical materials, such as the refractive index and thermal expansion coefficient, are commonly temperature-dependent [34,35]. We therefore investigated the thermal distribution of the lens to ensure that the optical liquid was not overheated. The simulation calculated the Joule heating but ignored the deformations of air thermal expansion. The 3D structure was entirely modeled using SolidWorks and exported into COMSOL (Figure 6a). Different electric potentials were applied to the electrodes of the resistance wire. The conductive direct-current model and the heat transfer module were adopted to define the boundary conditions shown in Figure 6b. The governing equations relating the heater transfer rate and temperature gradient include the thermal conduction and thermal convection in solids and fluidics.

Figure 6c shows the microheater structure and encircling air chamber arranged symmetrically around the optical liquid, which ensures that the temperature distribution is homogeneous across the horizontal direction of the fluid. The heater structure heats the air sealed in the chamber but is not in direct contact with the optical liquid. The air is an excellent thermal insulator (κ ≈ 0.025 W/mK), and the air cavities thus reduce the thermal gradient within the optical liquid. Moreover, the optical liquid is inserted into the buried microchannel without direct contact with the glass substrate, such that the thermal gradient in the vertical visual pathway is actively minimized. Along the optical path (Figure 6d), the temperature variation in the liquid is less than 0.2 °C in the horizontal direction and 0.4 °C in the vertical direction.

## 3. Fabrication and Assembly

### 3.1. D-Printed Microfluidic Chip

**Figure 7:**
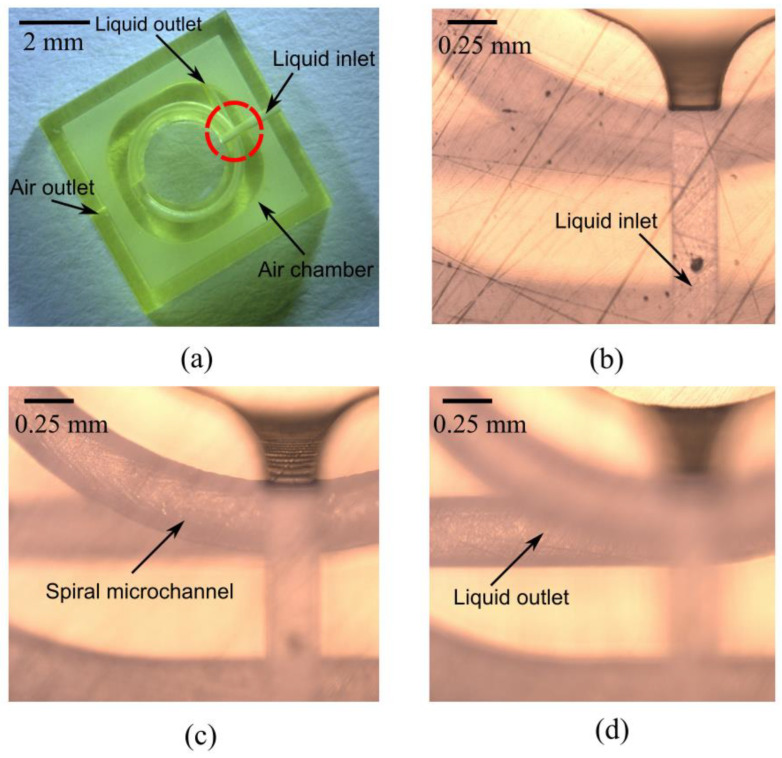
(**a**) Photograph of the device fabricated through 3D printing. (**b**–**d**) Optical microscope images of the structure within the red dashed circles presented in (**a**). Note that only a region of 1.5 × 1.5 mm^2^ on the top of the microfluidic chip is measured, owing to the limited field range of the microscopy.

The 3D optofluidic chip was fabricated using a commercial 3D printer (nanoArch P140, BMF Precision Technology Co., Ltd., Shenzhen, China). The technology is based on projection micro-stereolithography technology with a precision of 10 μm in the xy-plane and z-direction. The 3D structure was designed using SolidWorks software. The CAD file was converted into a standard triangulation language (STL) file, which was digitally sliced into individual layers and sequentially realized to build the 3D structure in a layer-by-layer manner. Figure 7a is a photograph of the completed 3D-printed structure. Figure 7b–d presents microscope photographs of the device that has spiral microchannels with circular cross-sections. The trajectory of the microchannels is outside of a single plane. The structure comprises a multilevel channel, with the first layer being for the liquid inlet, the second layer for the spiral fluid transportation, and the third layer for the liquid outlet.

### 3.2. Lens Assembly

**Figure 8:**
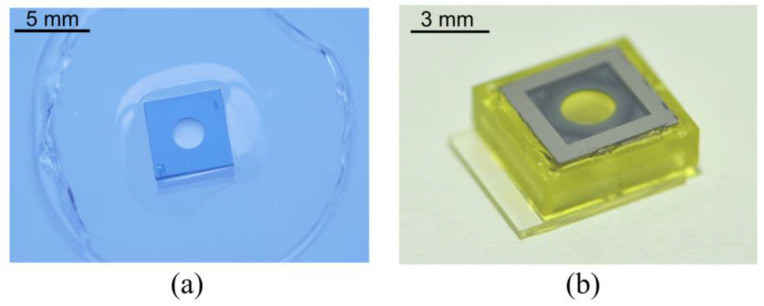
Photographs of (**a**) the polyacrylate membrane suspended on the silicon lens chip and (**b**) optofluidic lens device. The membrane is sandwiched between the silicon lens chip and the 3D-printing device.

Polyacrylate elastomer tape (VBH4905, 3M) was used as the optical membrane for its elasticity, transparency, and self-stickiness. A chamber comprised a cylindrical space and a tube connected to a vacuum pump. Before fixing the tape onto the device, a silicon lens chip was aligned with the center. A negative pressure deformed the membrane to become a hemispherical surface that was attached firmly to the silicon chip. After a few seconds, the remaining parts of the bubble were cut away mechanically, leaving the stretched film on the silicon surface (Figure 8a). The characteristics of the membrane were discussed in greater detail in our previous work [36].

Figure 8b is a photograph of the lens device. The silicon chip was fabricated by standard silicon bulk micromachining. The process combined KOH wet chemical etching and DRIE dry etching of a 4-inch, 300 μm-thick, (100) orientated silicon wafer. Silicon chip was chosen as the substrate of the thin film, because its smooth surface has a good bonding effect with the membrane. Black resin has worked for optical system packages, such as endoscopes, but the 3D-printing material we used here is high transparency in the visible spectrum, so the silicon chip also functions as a diaphragm in the liquid lens. Commercially available ITO film glass (0.5 mm in thickness) was used as a substrate to provide mechanical support and the electrical thermal actuator. The sheet resistance was 7–10 Ω/sq, and the thickness of the ITO film was approximately 150 nm. The ITO film was structured through laser etching (Luoyang Shangzhuo Technology Co., Ltd., Luoyang, China).

**Figure 9:**
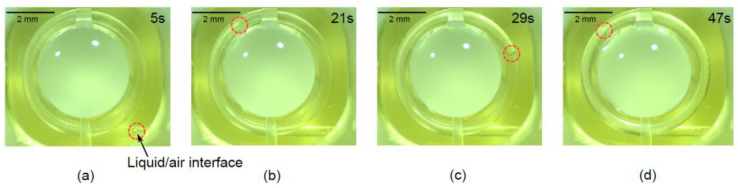
(**a**–**d**) Liquid flowing in the 3D spiral microchannel at different moments. A buried 3D spiral microchannel connects the air chamber and liquid chamber directly. Two light spots on the liquid are the reflected images of the light-emitting-diode illumination of the microscope used. The dynamic flowing in the spiral microchannel is presented as a Video S1 in Supplementary Materials.

The optical liquid was first filled from the liquid inlet with a micro-needle in the final assembly step. After filling the optical liquid into the lens chamber, an ultraviolet (UV)-curable epoxy was applied to seal the fluid inlet permanently. Then, the ITO heater chip was glued with the 3D-printed microfluidic chip using UV glue. As the last opening for keeping the pressure balance, the air outlet was sealed with UV glue, and the liquid lens package was complete. It is easy to package the device using ultraviolet-curable epoxy, because the 3D microchannel is embedded in the printing structure. The small amount of adhesive does not bury the microchannels. Figure 9 presents the liquid flowing in the microchannel when a voltage of 6 V was applied to the heater.

## 4. Measurement and Discussion

### 4.1. Thermal-Pneumatic Actuator Characteristics

**Figure 10:**
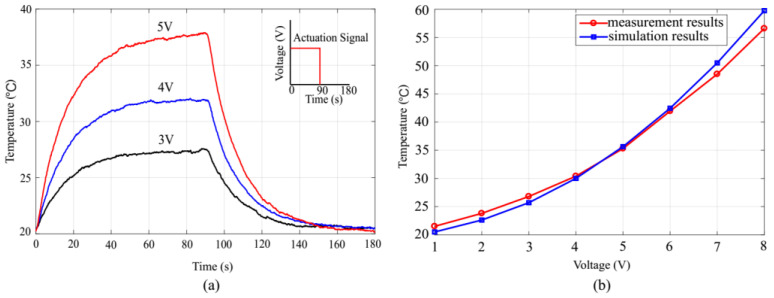
(**a**) Transient behavior of the thermal actuator during the heating and cooling process under different voltages (3, 4, and 5 V) applied for 90 s. The heat dissipated through natural heat convection. (**b**) Measurements and simulation results of the temperature under different voltages. The temperature is proportional to the voltage squared.

We measured the dynamic temperature change of the actuator when the voltage was first applied at 0 s and when it was cut off at 90 s using the infrared thermal imager (FLIR, ETS320). As shown in Figure 10a, the temperature increased rapidly when the current was applied with a time constant of approximately 15 s, and the temperature reached 95% of the full-range steady state needed after an additional approximately 25 s. The temperature then increased slowly and eventually approached and maintained a stable temperature within 90 s. Upon turning off the current, the temperature initially dropped sharply and then returned gradually to the ambient temperature within 60 s.

Figure 10b shows the relationship between the stable temperature of the thermal actuator and the applied voltage. The temperature was measured under the thermal equilibrium condition using a minuscule thermocouple temperature sensor, which was inserted into the air chamber from the air outlet and sealed using a tiny droplet of ultraviolet glue. The results are in good agreement with the simulation results obtained using the COMSOL software.

### 4.2. Optical Characteristics

**Figure 11:**
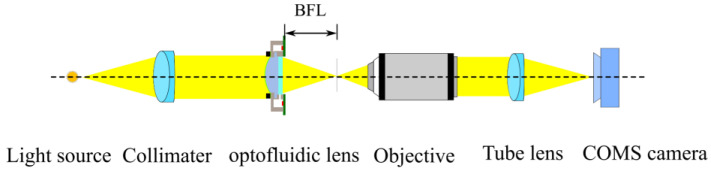
Diagram of the BFL measurement setup.

**Figure 12:**
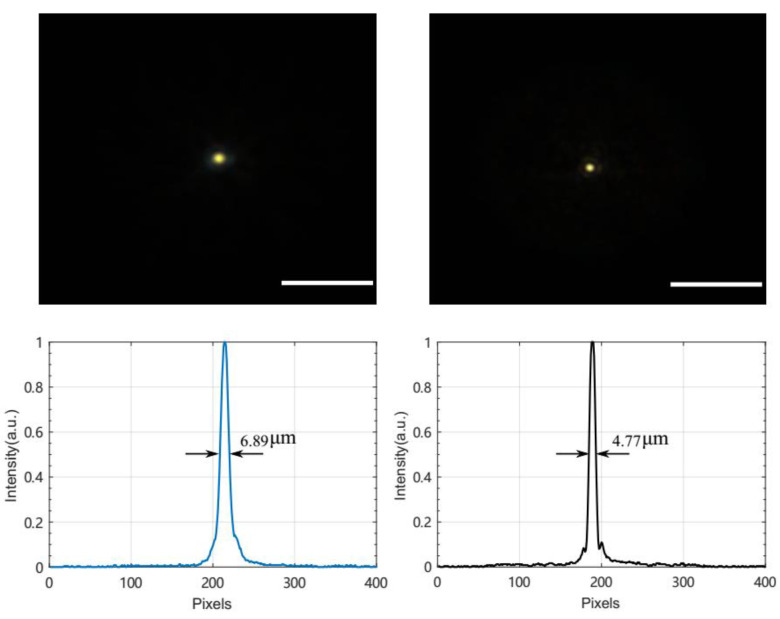
Focusing characteristics of the optofluidic lens under voltages of 2.5 and 5.5 V. Scale bars: 50 μm.

An infinity-corrected optical microscope was used to measure the back focal length (BFL) of the lens (Figure 11). A multimode fiber with a diameter of 100 μm was used to approximate a point light source. The emitted light beam was collimated and entered the optofluidic lens from the convex surface, and it was then focused at the focal plane of an infinity-corrected 10 × microscope objective (FL:18 mm, Olympus, Japan). An achromatic tube lens (FL:180 mm, Olympus, Japan) was applied to image the focused spot onto a complementary metal–oxide–semiconductor (CMOS) camera (acA2440, BASLER, Ahrensburg Germany). The objective was fixed on an electric moving stage (MTS50, Thorlab, Newton, NJ, USA). By moving the objective to observe the image of the focused beam, the point at which the focused circular image on the CMOS camera had the smallest possible size was found, and the BFL was determined. Figure 12 shows optical microscopy images of the focal spots of the optofluidic lens under different voltages.

**Figure 13:**
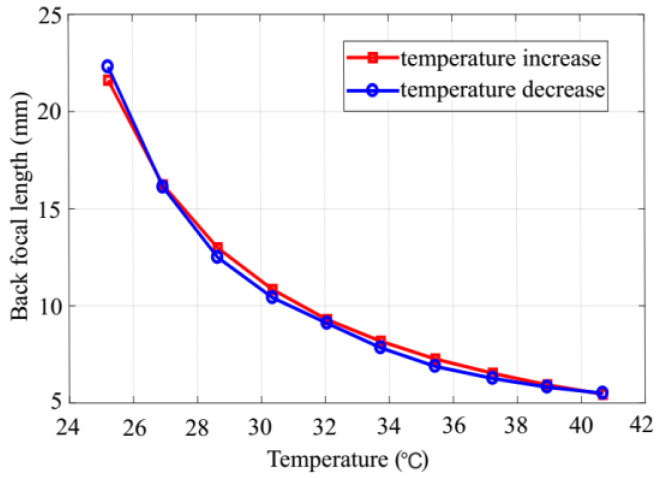
Relationship between the temperature and BFL. The focal length was measured as the operating temperature was increased from 25 to 40 °C and then decreased again to 25 °C. The two curves overlap well and show that the optical membrane has high elasticity without noticeable hysteresis.

The BFL was measured as a function of the thermal actuator temperature (Figure 13). The temperature was increased from 25 to 40 °C to find the focal length as a function of the temperature. The results show that the focal length decreased from 22.5 to 5.5 mm and the numerical aperture increased from 0.08 to 0.18 accordingly. The maximum voltage was lower than 6 V, corresponding to power consumption of 120 mW.

**Figure 14:**
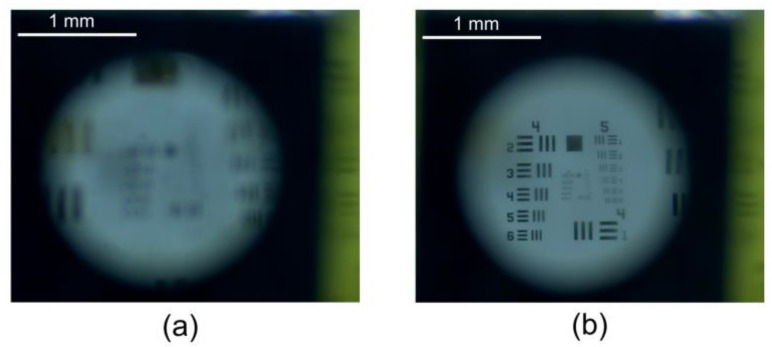
Captured images of a 1951 USAF resolution test chart when voltages of (**a**) 0 V and (**b**) 5.5 V were applied to the lens.

Figure 14 presents two snapshots of an imaged 1951 USAF resolution test chart. The CMOS camera with a macro lens was used to record the image. The focal length of the macro camera was fixed to ensure that the camera did not contribute to focusing. Before the voltage was applied, the observed image was out of focus, and the image was blurry (Figure 14a). The image was focused and a stable magnified image was observed at a high voltage of 5.5 V applied on the lens (Figure 14b). The imaging aberration can be explained, since the membrane deformation does not have an ideal spherical or parabolic surface, and the less-desirable aspheric curvature will reduce the image quality. In addition, the uneven stress distribution of the membrane and the inflection point of the membrane profile near the aperture edge give rise to spherical aberration.

Table 1 compares the performance of different thermo-actuated lenses. The on-chip thermo-pneumatically tunable liquid lens can be used for minimally invasive biomedical applications [29]. Thermo-pneumatic pumps generate larger volume displacements than piezoelectric actuation but with limited stroke frequency due to slow cooling periods. The choice of an appropriate actuation mechanism depends on the application. For low voltages or low power consumption, thermo-pneumatic systems are preferable, and they are also preferable for stable and precise fine-tuning of small flow rates at low frequencies [37]. A thermo-electric (TE) device was integrated into a liquid chamber, resulting in a more responsive tunable liquid microlens with a temperature response time of less than 1s. However, the device packaging needs to be further improved [38].

Except for liquid materials, PDMS material with a large thermal expansion coefficient can be used to fabricate solid tunable lenses [39]. Solid tunable lenses can more readily withstand temperature, pressure, and motion fluctuations than liquid lenses. Nevertheless, a high temperature and large power consumption are required to change the lens curvature—the small volume expansion of solid material results in a low dynamic focal length. 

The convenient optofluidic chip fabrication is based on a one-step molding method. It molds the structure features on a photolithographic resist and pattern with the PDMS material. Then, the PDMS structure follows by sealing the open channels with a flat substrate and finally creating rectangular cross-section channels. The one-step molding is very conventional, but it is limited to fabricating a 3D optofluidic lens structure, because multilayer channels will be interlocked. Here, we used 3D printing to fabricate truly 3D microchannel networks, which solves the issue of liquid leakage because the microchannels are embedded in the structure. Compared with the one-step molding fabrication, the 3D printing offers several significant advantages: it does not require a clean-room environment; it can fabricate different microchannel profiles other than rectangular channels in photolithography; it is straightforward to fabricate 3D structures; it does not involve any alignment or sacrificial part; PDMS no longer limits chip material, and the material can be UV-printable resin, which has higher mechanical hardness and stability.

## 5. Conclusions

We reported the design and fabrication of a miniature optofluidic microlens with an encircling thermal-pneumatic actuator. The 3D-printing fabrication process achieves a truly 3D microfluidic chip with high efficiency and low cost. Compared with photolithography, the 3D-printed microfluidic chip for the optofluidic lens has the advantages of actual 3D capability, applicability to various optical liquids, and a simple procedure. The tuning of an optofluidic lens was demonstrated using a thermo-pneumatic actuator that pumps optical liquid into the closed microfluidic channels. Continuous tuning of the focal length over a wide range was achieved by applying different voltages. The method has the advantages of simplicity, a low power voltage, excellent repeatability owing to the microfluidic network, and there being no need for external mechanical moving parts. Minimal thermal cross-talk between the thermal air chamber and the optical liquid was realized through finite-element simulation. The proposed optofluidic lens has numerous potential applications, including fine-tuning microscopy imaging and biomedical in situ testing.

## Figures and Tables

**Table 1:** Comparison of thermal actuated tunable lenses.

Lens Type	Expansion	Actuation	Device Size, mm^3^	Aperture Size, mm	Focal Length, mm	Temperature	Response Time, s	Ref:
M-L lens	Air	Thermo-pneumatic *	12 × 12 × 1.2	2	∞~5	25~48 °C	N/R	[29]
M-L lens	Air	Thermo-pneumatic *	6 × 6 × 2	2	∞~5	25~40 °C	45	This work
M-L lens	Air	Thermo-pneumatic *	N/R	2	∞~4	20~48 °C	30	[37]
L-L lens	Liquid	Thermo-electric(TE)	N/R	2	−80~−20	20~30 °C	0.8	[38]
Solid lens	Solid	Joule heating *	1.5 × 1.5 *	0.3	1.0~1.9	50~350 °C	N/R	[39]

N/R: Not report; M-L lens: Membrane–liquid lens; L-L lens: Liquid–liquid lens. * Actuator-in-device; 1.5 × 1.5 * estimated from SEM image.

## Data Availability

Not applicable.

## Supplementary Materials

The following supporting information can be downloaded at: https://www.mdpi.com/article/10.3390/mi13081189/s1, Video S1: The dynamic flowing in the spiral microchannel.

## References

[1] Kim W., Yang H.C. (2017). Wide and fast focus-tunable dielectro-optofluidic lens via pinning of the interface of aqueous and dielectric liquids. Opt. Express.

[2] Bianco V., Mandracchia B., Marchesano V., Pagliarulo V., Olivieri F., Coppola S., Paturzo M., Ferraro P. (2017). Endowing a plain fluidic chip with micro-optics: A holographic microscope slide. Light Sci. Appl..

[3] Huang H., Zhao Y. (2019). Optofluidic lenses for 2D and 3D imaging. J. Micromech. Microeng..

[4] Zhao P., Ataman Ç., Zappe H. (2016). Gravity-immune liquid-filled tunable lens with reduced spherical aberration. Appl. Opt..

[5] Park I.S., Park Y., Oh S.H., Yang J.W., Chung S.K. (2018). Multifunctional liquid lens for variable focus and zoom. Sens. Actuators A Phys..

[6] Beeckman J., Yang T.H., Nys I., George J.P., Lin T.H., Neyts K. (2018). Multi-electrode tunable liquid crystal lenses with one lithography step. Opt. Lett..

[7] Wang Z., Qu W., Yang F., Tian A., Asundi A. (2017). Absolute measurement of aspheric lens with electrically tunable lens in digital holography. Opt. Lasers Eng..

[8] Lee J., Park Y., Chung S.K. (2019). Multifunctional liquid lens for variable focus and aperture. Sens. Actuators A Phys..

[9] Li L., Wang J.H., Wang Q.H., Wu S.T. (2018). Displaceable and focus-tunable electrowetting optofluidic lens. Opt. Express.

[10] Cheng C.C., Chang C.A., Yeh J.A. (2006). Variable focus dielectric liquid droplet lens. Opt. Express.

[11] Lopez C.A., Hirsa A.H. (2008). Fast focusing using a pinned-contact oscillating liquid lens. Nat. Photon..

[12] Nguyen N.T. (2010). Micro-optofluidic lenses: A review. Biomicrofluidics.

[13] Tang S.K.Y., Stan C.A., Whitesides G.M. (2008). Dynamically reconfigurable liquid-core liquid-cladding lens in a microfluidic channel. Lab Chip.

[14] Seow Y.C., Liu A.Q., Chin L.K., Li X.C., Huang H.J., Cheng T.H., Zhou X.Q. (2008). Different curvatures of tunable liquid microlens via the control of laminar flow rate. Appl. Phys. Lett..

[15] Chin L.K., Liu A.Q., Lim C.S., Lin C.L., Ayi T.C., Yap P.H. (2010). An optofluidic volume refractometer using Fabry–Pérot resonator with tunable liquid microlenses. Biomicrofluidics.

[16] Mao X.L., Lin S.C.S., Lapsley M.I., Shi J.J., Juluri B.K., Huang T.J. (2009). Tunable Liquid Gradient Refractive Index (L-GRIN) lens with two degrees of freedom. Lab Chip.

[17] Xiong K.D., Yang S.H., Li X.W., Xing D. (2018). Autofocusing optical-resolution photoacoustic endoscopy. Opt. Lett..

[18] Dai B., Jiao Z.A., Zheng L.L., Bachman H., Fu Y.F., Wan X.J., Zhang Y.L., Huang Y., Han X.D., Zhao C.L. (2019). Colour compound lenses for a portable fluorescence microscope. Light Sci. Appl..

[19] Fuh Y.K., Lin M.X., Lee S. (2012). Characterizing aberration of a pressure-actuated tunable biconvex microlens with a simple spherically-corrected design. Opt. Lasers Eng..

[20] Zhou H., Zhang X., Xu Z., Wu P., Yu H. (2019). Universal membrane-based tunable liquid lens design for dynamically correcting spherical aberration over user-defined focal length range. Opt. Express.

[21] Waibel P., Mader D., Liebetraut P., Zappe H., Seifert A. (2011). Chromatic aberration control for tunable all-silicone membrane microlenses. Opt. Express.

[22] Gowda H.G.B., Wallrabe U. (2019). Simulation of an Adaptive Fluid-Membrane Piezoelectric Lens. Micromachines.

[23] Farghaly M.A., Akram M.N., Halvorsen E. (2016). Modeling framework for piezoelectrically actuated MEMS tunable lenses. Opt. Express.

[24] Ren H., Fox D., Anderson P.A., Wu B., Wu S.T. (2006). Tunable-focus liquid lens controlled using a servo motor. Opt. Express.

[25] Shian S., Diebold R.M., Clarke D.R. (2013). Tunable lenses using transparent dielectric elastomer actuators. Opt. Express.

[26] Deng K., Rohn M., Gerlach G. (2016). Design, simulation and characterization of hydrogel-based thermal actuators. Sens. Actuators B Chem..

[27] Handique K., Burke D.T., Mastrangelo C.H., Burns M.A. (2001). On-chip thermopneumatic pressure for discrete drop pumping. Anal. Chem..

[28] Chia B.T., Liao H.-H., Yang Y.-J. (2011). A novel thermo-pneumatic peristaltic micropump with low temperature elevation on working fluid. Sens. Actuators A Phys..

[29] Zhang W., Zappe H., Seifert A. (2014). Wafer-scale fabricated thermo-pneumatically tunable microlenses. Light Sci. Appl..

[30] Waheed S., Cabot J.M., Macdonald N.P., Lewis T., Guijt R.M., Paull B., Breadmore M.C. (2016). 3D printed microfluidic devices: Enablers and barriers. Lab Chip.

[31] Hu Y., Rao S., Wu S., Wei P., Qiu W., Wu D., Xu B., Ni J., Yang L., Li J. (2018). All-glass 3D optofluidic microchip with built-in tunable microlens fabricated by femtosecond laser-assisted etching. Adv. Opt. Mater..

[32] Saggiomo V., Velders A.H. (2015). Simple 3D printed scaffold-removal method for the fabrication of intricate microfluidic devices. Adv. Sci..

[33] Olanrewaju A., Beaugrand M., Yafia M., Juncker D. (2018). Capillary microfluidics in microchannels: From microfluidic networks to capillaric circuits. Lab Chip.

[34] Berto P., Philippet L., Osmond J., Liu C.F., Afridi A., Marques M.M., Agudo B.M., Tessier G., Quidant R. (2019). Tunable and free-form planar optics. Nat. Photon..

[35] Zhang H., Ren H., Xu S., Wu S.T. (2014). Temperature effects on dielectric liquid lenses. Opt. Express.

[36] Zhang W., Zappe H., Seifert A. (2013). Polyacrylate membranes for tunable liquid-filled microlenses. Opt. Eng..

[37] Lee J.K., Park K.W., Lim G.B., Kim H.R., Kong S.H. (2012). Variable-focus liquid lens based on a laterally-integrated thermopneumatic actuator. J. Opt. Soc. Korea.

[38] Ashtiani A.O., Jiang H.R. (2013). Thermally actuated tunable liquid microlens with sub-second response time. Appl. Phys. Lett..

[39] Lee S.Y., Tung H.W., Chen W.C., Fang W.L. (2006). Thermal Actuated Solid Tunable Lens. IEEE Photon. Technol. Lett..

